# Sent‐Induced Coronary Stenosis in a Previously Noncritical Segment Following PCI: A Case Report

**DOI:** 10.1002/ccr3.72555

**Published:** 2026-04-14

**Authors:** Hamzeh Habibi, Soroush Mostafavi

**Affiliations:** ^1^ Department of Cardiology, Hazrat‐e Rasool General Hospital, School of Medicine Iran University of Medical Sciences (IUMS) Tehran Iran

**Keywords:** acute coronary syndrome, coronary angioplasty, hypersensitivity reaction, in‐stent restenosis, stenting

## Abstract

While percutaneous coronary interventions (PCI) with stenting are standard in treating coronary artery disease, rare complications such as stent‐induced de novo stenosis can paradoxically provoke acute coronary syndromes (ACS). We present the case of a 48‐year‐old female (Middle Eastern descent) with a recent history of coronary angioplasty, who presented with ACS 2 months post‐procedure. Coronary angiography revealed critical stenosis in the left main (LM), a previously noncritical segment. Additionally, there was significant in‐stent restenosis in the previously deployed stents in the left anterior descending (LAD) and left circumflex (LCX) arteries. This recurrent scenario of stenosis development in non‐stented regions following stent placement suggests the need to explore underlying hypersensitivity reactions as one of several possible mechanisms. Further investigation into the mechanisms, whether immunological or atherosclerotic, is warranted.

## Key Message

This case highlights an unusual complication of coronary stenting, where severe stenosis developed not only in previously treated segments but also in a normal adjacent artery (left main) shortly after PCI. It raises concern about hypersensitivity reactions or local inflammatory responses as potential mechanisms, warranting further investigation and close post‐PCI monitoring.

## 1 Introduction

Percutaneous coronary intervention (PCI) with stent implantation is a cornerstone in the management of coronary artery disease (CAD). Despite its success, PCI is associated with complications such as in‐stent restenosis (ISR) and stent thrombosis, which may lead to significant morbidity. While ISR is a well‐documented complication of PCI, the development of rapid, de novo stenosis in previously angiographically noncritical, non‐stented coronary segments, particularly in the LM artery, remains exceptionally rare and poorly understood. This phenomenon challenges conventional models of atherosclerosis progression and raises concerns about paracrine or systemic inflammatory responses triggered by stent implantation. Such cases carry high clinical stakes due to the potential for sudden hemodynamic collapse and limited therapeutic options, underscoring the need for heightened vigilance and further mechanistic investigation.

## 2 Case History/Examination

A 48‐year‐old woman of Middle Eastern descent with a history of angioplasty and coronary stenting presented with nausea, vomiting, and acute chest pain a few hours before admission. She reported exertional chest discomfort over the past 2 months, consistent with effort‐induced angina. However, the chest pain became more severe 3 h prior to admission, accompanied by diaphoresis and dyspnea. Initial assessment in the emergency room revealed hyperglycemia (420 mg/dL) and metabolic acidosis, leading to consideration of diabetic ketoacidosis (DKA); however, due to persistent symptoms, cardiology consultation was sought.

**FIGURE 1:**
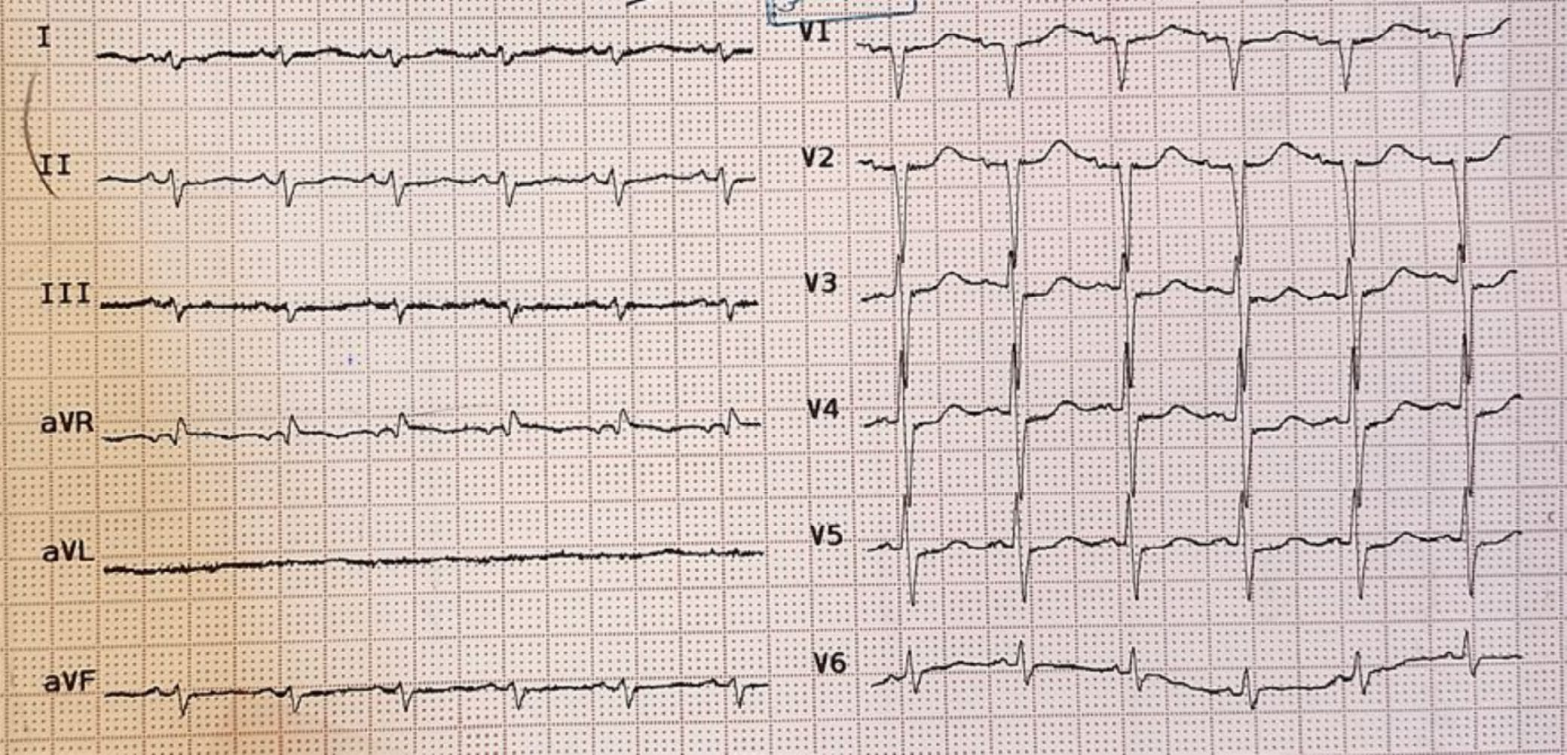
ECG showed sinus tachycardia, left axis deviation, low‐voltage QRS complexes, and nonspecific ST‐T changes.

Upon examination, the patient appeared distressed, with vital signs indicating a heart rate of 130 beats per minute, systolic blood pressure of 80 mmHg, respiratory rate of 30 breaths per minute, and oxygen saturation of 90% on ambient air. The dyspnea was orthopneic in nature, improving in an upright posture. Chest X‐ray revealed increased pulmonary vasculature suggestive of pulmonary edema, while electrocardiography (ECG) revealed sinus tachycardia (heart rate: 130 bpm), left axis deviation, and low voltage QRS complexes along with nonspecific ST‐T abnormalities (Figure 1).

Subsequent echocardiography demonstrated severe left ventricular dysfunction with an ejection fraction (EF) of 25%, while the right ventricle appeared normal with no evidence of pericardial effusion. Despite treatment for acute decompensated heart failure (ADHF), dyspnea persisted, and hemodynamics remained unstable. Differential diagnoses including pulmonary thromboembolism (PTE), aortic dissection, and cardiac tamponade were excluded based on clinical and paraclinical assessments.

## 3 Differential Diagnosis, Investigations, and Treatments

**FIGURE 2:**
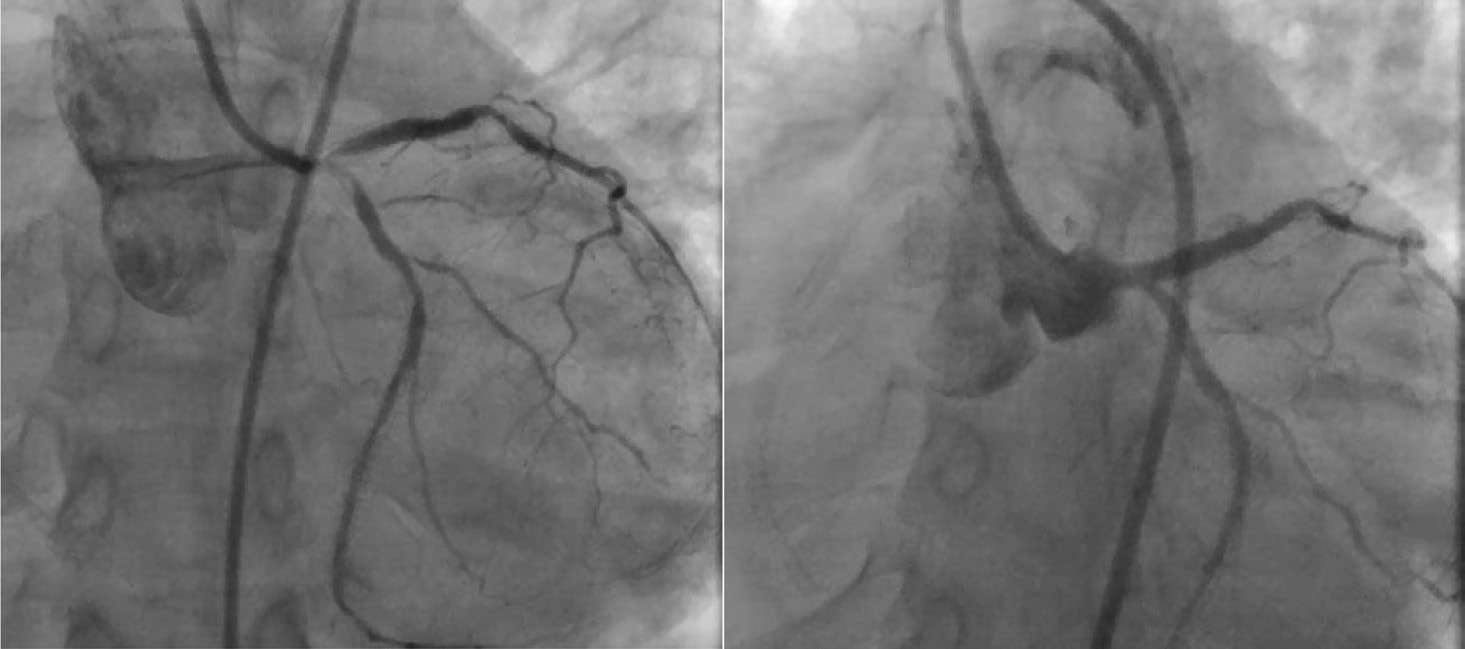
Coronary angiography revealed critical stenosis in the left main (LM) artery alongside subtotal occlusion of both the left anterior descending (LAD) and left circumflex (LCX) arteries. Consequently, successful angioplasty was performed on the LM artery as well as the LAD and LCX arteries.

Despite an initial negative serum troponin level, the patient was hemodynamically unstable, with persistent hypotension, sinus tachycardia, and worsening pulmonary edema in the setting of newly documented severe left ventricular systolic dysfunction (LVEF 25%). The clinical picture was consistent with cardiogenic shock secondary to acute coronary syndrome. Angiography revealed significant stenosis in the LM and subtotal occlusion of the left anterior descending (LAD) and left circumflex (LCX) arteries (Figure 2). Given the patient's unstable hemodynamic status, immediate LM angioplasty, along with intervention on the LAD and LCX, was successfully conducted.

**FIGURE 3:**
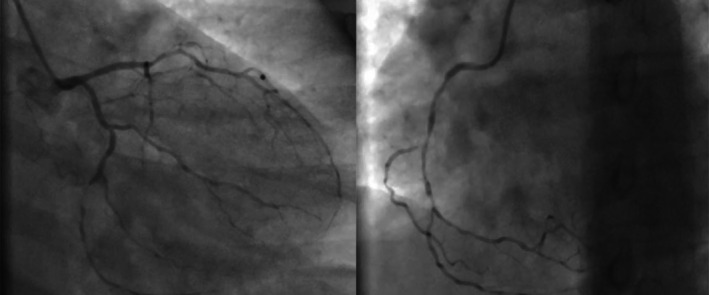
On January 7, 2023, the initial angiography was performed in response to typical chest pain, revealing triple‐vessel disease (3VD).

**FIGURE 4:**
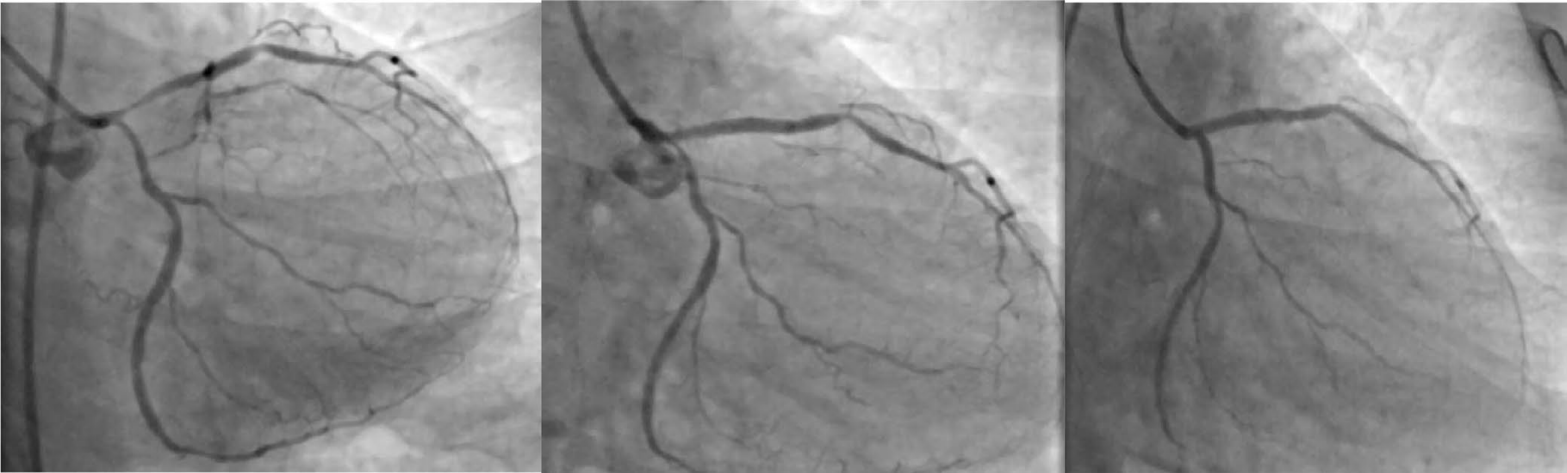
A follow‐up coronary angiography was conducted just 2 months later, on March 1, 2023, (left panel) in response to new chest pain, revealing significant stenosis in the left anterior descending (LAD) artery and moderate stenosis in the left circumflex (LCX) artery. Consequently, LAD stenting was performed. Another angiography was performed on May 10, 2023, due to continued chest pain, revealing that the LCX proximal stenosis had progressed to 90% (middle panel). As depicted in the right image, the LCX was stented from the ostium to the proximal part using a third‐generation drug‐eluting stent (DES), specifically the Ultimaster 2.5 × 15 mm (right panel).

A review of previous angiography and angioplasty videos indicated a history of multivessel coronary artery disease (CAD) 6 months prior, prompting consideration of coronary artery bypass grafting (CABG), which was declined by the patient (Figure 3). Subsequently, a multivessel percutaneous coronary intervention (PCI) strategy was pursued, involving stenting of the LAD and LCX during a single session. However, within 2 months, recurrent chest pain led to repeat coronary angiography, revealing significant stenosis in previously noncritical segments of the LAD and LCX, necessitating additional stenting procedures (Figure 4).

## 4 Outcome and Follow‐Up

During the current hospitalization on July 19, 2023, the patient experienced a recurrence of symptoms, including more severe chest pain, leading to hypotension and shock. Coronary angiography demonstrated severe stenosis in the previously noncritical LM, as well as subtotal in‐stent restenosis in the recently treated LAD and LCX arteries. The LM was treated with high‐pressure balloon angioplasty alone due to distal tapering and concern for ostial LAD or LCX compromise. Due to resource constraints and hemodynamic instability, intravascular imaging including IVUS or OCT was not performed during any procedure.

Following the procedure, the patient was transferred to the cardiac care unit (CCU) for further medical management. After 4 days of hospitalization, the patient was discharged in stable condition. A concise timeline of the patient's clinical course is summarized in the accompanying table (Table 1).

**TABLE 1:** Timeline table with details of interventions performed.

Date	Event	Findings/intervention
January 2023	Diagnostic angiography	3‐vessel CAD
February 2023	PCI #1	LAD: Synergy (3.0 × 28 mm, 11 atm) and LCx: Ultimaster (2.5 × 15 mm, 12 atm)
April 2023	PCI #2 (for recurrent angina)	Additional stents in distal LAD (Xience 3 × 18 mm, 12 atm) and mid‐LCx (Xience 2.75 × 18 mm, 12 atm)
July 19, 2023	PCI #3 (emergency admission)	Cardiogenic shock; de novo critical LM stenosis; bailout balloon angioplasty of LM, LAD (Onyx 2.75 × 18 mm, 12 atm and Supreme 3 × 20 mm, 11 atm), LCx (Onyx 2.5 × 26 mm, 12 atm)

## 5 Discussion

ISR is defined as > 50% luminal narrowing within the stented segment, typically presenting as unstable angina or, less commonly, myocardial infarction [1]. The presence of metal stents may trigger local inflammation, vascular remodeling, and endothelial dysfunction [2].

Four mechanisms have been proposed for drug‐eluting stent (DES) restenosis: mechanical, biological, genetic, and technical factors [3]. Within biological factors, drug resistance and allergic inflammatory responses to polymer or metal scaffolds play crucial roles [4]. The inflammatory cascade triggers local neointimal proliferation [5]. Approximately 17% of the population is allergic to nickel, with 1%–2% exhibiting allergies to other stent materials like cobalt and chromium [6].

New‐generation DESs employ metal alloys with thinner struts and reduced nickel content, such as cobalt‐chromium (CoCr) and platinum‐chromium (PTCR) stents. Despite improvements in stent design, nickel hypersensitivity remains a significant contributor to ISR in cobalt‐chromium stents [7].

Previous reports highlight frequent ISR occurrences post‐CoCr stent placement in patients with confirmed nickel allergies. However, prognosis markedly improves following the adoption of absorbable vascular stents and adjunctive prednisolone and anti‐allergic therapies post‐PCI [8].

Our case presents two unique features: early onset of stent restenosis within 2 months and involvement of both stented and previously noncritical segments. Hypersensitivity reactions, as possible mechanisms, may extend beyond the stented zone, contributing to stenosis in neighboring segments. Histopathological analyses of explanted stents from patients with recurrent ISR have demonstrated lymphocytic infiltration, eosinophilic accumulation, and fibrinoid necrosis findings consistent with type IV delayed hypersensitivity reaction [8].

In this case, attention should also be paid to the more common mechanisms of restenosis. These mechanisms include incomplete stent dilation, incomplete lesion coverage, improper stent placement, edge dissection, and aggressive progression of primary atherosclerotic disease. The lack of intravascular imaging limits our ability to differentiate between these causes. This case raises awareness of the rare but possible complication of stenting‐related stenosis in adjacent non‐stented coronary segments, underscoring the need for heightened vigilance and further mechanistic studies.

## Author Contributions

**Hamzeh Habibi:** conceptualization, methodology, supervision, writing – review and editing. **Soroush Mostafavi:** conceptualization, data curation, writing – original draft, writing – review and editing.

## Funding

The authors have nothing to report.

## Consent

Written informed consent was obtained from the patient. A copy of the written consent is available.

## Conflicts of Interest

The authors declare no conflicts of interest.

## Data Availability

Data available on request from the authors.
